# Risk Profile of Bacteriophages in the Food Chain

**DOI:** 10.3390/foods14132257

**Published:** 2025-06-26

**Authors:** Monika Trząskowska, Eyesun Eedo Naammo, Muhammad Salman, Ayomide Afolabi, Catherine W. Y. Wong, Danuta Kołożyn-Krajewska

**Affiliations:** 1 Department of Food Gastronomy and Food Hygiene, Institute of Human Nutrition Sciences, Warsaw University of Life Sciences, Nowoursynowska Str. 159C, 02-776 Warsaw, Poland; 2 Institute of Food Science, Faculty of the Agricultural and Food Sciences and Environmental Management, University of Debrecen, Böszörményi Str. 138, H-4032 Debrecen, Hungary; eyesun.naammo@uod.ac; 3 Department of Advanced Nano-Medical Engineering, Institute of Science Tokyo, 1-5-45 Yushima, Bunkyo-ku, Tokyo 113-8510, Japan; salman.muhammad@tmd.ac.jp; 4 Faculty of Human Nutrition, Warsaw University of Life Sciences, Nowoursynowska Str. 159C, 02-776 Warsaw, Poland; ayoafolabi111@gmail.com; 5 Institute for Food Safety and Health, Department of Food Science and Nutrition, Illinois Institute of Technology, 6502 S Archer Rd., Bedford Park, IL 60501, USA; cwong21@iit.edu; 6 Department of Dietetics and Food Research, Jan Długosz University in Częstochowa, Armii Krajowej Ave. 13/15, 42-200 Częstochowa, Poland; d.kolozyn-krajewska@ujd.edu.pl

**Keywords:** bacteriophages, biocontrol, bacteriophage application risks, food chain, safety, phage-mediated gene transfer, risk profile

## Abstract

Phages are considered effective biocontrol agents for improving food safety due to their specific interaction with pathogens. It is essential to recognise that zero risk does not exist, and as biological agents, phages must be continuously evaluated for potential adverse effects on human health in both food and clinical contexts. This is the first bacteriophage risk profile performed according to the methodology recommended by FAO/WHO and EFSA. Key safety concerns regarding phage use in the food sector include the risk of horizontal gene transfer, especially regarding antibiotic resistance genes among bacteria. While such occurrences are contextually dependent and rare, they warrant further scrutiny. Moreover, improper phage application during food processing could lead to the emergence of resistant bacterial strains, compromising the long-term efficacy of phage interventions. Currently, there is limited evidence indicating any health risks linked to phage consumption or pathogenic behaviour (e.g., possible association between bacteriophages and Parkinson’s disease). Despite numerous studies affirming the safety and efficacy of phages in the food chain, continuous monitoring remains crucial. In particular, the responses of susceptible populations to phage exposure should be carefully examined.

## 1. Introduction

Bacteriophages (phages) are viruses with the unique capacity to infect and spread throughout bacteria. They have a complex structure that includes a protein covering their genetic material, which may occur in the form of DNA or RNA [1].

In recent years, phages have garnered considerable attention as promising biocontrol agents for enhancing food safety due to their high specificity toward target pathogens. Unlike broad-spectrum antimicrobials, phages can selectively eliminate specific strains (e.g., *Listeria monocytogenes* or serotypes of *Salmonella enterica*) without disturbing the natural microbiota of food matrices [2,3]. Phages offer advantages over conventional methods, such as preserving nutritional properties, leaving no aftertaste, and being effective against antibiotic-resistant bacteria [4].

In the past five years, many studies have been conducted using phages to reduce pathogen populations inoculated on fresh produce and poultry to varying degrees. There is already regulatory approval for certain bacteriophage preparations in specific food applications. In the United States, the Food and Drug Administration (FDA) has granted “Generally Recognised As Safe” (GRAS) status to several phage products for use against foodborne pathogens such as *Listeria monocytogenes* in ready-to-eat meats, seafood, meat products, fish, and cheese. Other phage products target *Escherichia coli* in red meat parts and trim, and *Salmonella* in poultry, fish, shellfish, and fresh and processed fruits and vegetables [5]. In contrast, within the European Union, the use of bacteriophages in food is more restricted. At the same time, the European Food Safety Authority has evaluated the safety and efficacy of Listex P100 against *Listeria monocytogenes* in different ready-to-eat meat products [6,7]. Despite the current regulatory approvals, there are still more technological and regulatory obstacles to overcome, both in the therapeutic and alimentary bacteriophage usage [4,8].

Effective management of risks arising from microbial hazards is technically complex. Risk analysis, involving risk assessment, risk management, and communication, has been introduced as a new approach to evaluating and controlling hazards to help protect consumers’ health and ensure fair practices in the food trade. It could also facilitate the judgment of equivalence in food safety control systems [9]. In food safety assessments, the complete elimination of risk is unattainable. It means that the zero-risk assumption is unachievable, especially in microbial risk assessment [10]. This perspective emphasises the importance of proactive measures and scientific evaluation in ensuring food safety while recognising inherent uncertainties. Risk-based approaches aim to manage and mitigate risks through informed decision-making and prevention strategies [11].

Recent analyses of the human metagenome and the phagobiota proteome, alongside investigations utilising pertinent animal models, have indicated that bacteriophages could be specific to certain pathologies, particularly those linked to protein misfolding. Phages may directly interact with proteins and eukaryotic cells, which could lead to autoimmune and neurological illnesses [12]. Bacterial genomes contain prophages that can encode virulence factors that cause botulism, diphtheria, and infections from *Streptococcus*, *Escherichia coli*, and *Staphylococcus aureus* [13]. Moreover, they may also play a role in the evolution of pathogens and antibiotic resistance [14].

Similarly to the discussion on the safety perspectives of probiotics and their therapeutic interventions in certain at-risk population groups [15,16], the risk assessment of bacteriophages also needs to be considered and conducted.

This review aims to produce a risk profile of phages in the food chain—specifically, to provide contextual and background information relevant to a food/hazard combination so that risk managers can make decisions and, if necessary, take further action. The basic risk management question is as follows: Do phages in the food chain pose a risk to human health?

## 2. Methodology

The methodology of this review is based on the search and analysis of information from publications.

A risk profile comprises a systematic collection of information needed to make a risk management decision and determine whether a complete risk assessment is required. This may help to determine the structure of the risk assessment, fine-tune risk management questions, and assess the feasibility of a more comprehensive risk assessment [17].

Therefore, an in-depth examination of relevant publications from databases, including PubMed, Scopus, Science Direct, and Google Scholar, was performed. The inquiry focused on keywords such as bacteriophage, phage, food safety, food chain, risk profile, hazard, and illness. The titles and abstracts of the articles were analysed, and redundancies were removed. Selected sources of evidence included research articles, reviews, brief communications, book chapters, and, minimally, websites of reputable institutions.

To facilitate the development of a phage risk profile, a series of questions was posed in two key areas: “Hazard and Food” and “Evaluation of Adverse Health Effects”. 

## 3. Risk Profile

### 3.1. Hazard and Food—Hazard Identification

**What is the biology of bacteriophages?**

Phages possess a complex structure with a protein coat surrounding their genetic material, either DNA or RNA [1]. Unlike bacteria, bacteriophages typically lack conserved genes, such as 16S rRNA. However, some phages can obtain bacterial 16S rRNA genes through transduction. Broad-host-range, generalised transducing phages have been demonstrated to acquire entire 16S rRNA genes from different bacterial genera [18]. Phage genomes can be as little as 3.4 kb or as large as 500 kb. The mosaic nature of phage genomes means that each unique genome represents a distinct arrangement of shuffled genetic modules. This reorganisation of modules over billions of years of viral evolution can be compared to creating sentences using different words [19].

To identify and bind to specific receptors on bacterial surfaces, bacteriophages employ unique receptor-binding proteins (RBPs) in their tail structures [20]. The host range and specificity of phages are significantly influenced by these RBPs, which comprise tail fibres and spikes [21]. They inject their genetic material into the host cell by this attachment, taking over the bacterial machinery and manufacturing fresh phage particles. The host cell is lysed in the final phase, releasing many phages that can infect and multiply within other bacterial cells [22].

Since there is a lack of specific receptors for bacteriophages on eukaryotic cells, these viruses were, for a long time, considered to be neutral to animals and humans. However, studies of recent years provided clear evidence that bacteriophages can interact with eukaryotic cells, significantly influencing the functions of tissues, organs, and systems of mammals, including humans. Bacteriophages have been shown to affect the functions of several systems in mammals, including the immune, respiratory, nervous, gastrointestinal, reproductive, and urinary tracts. Moreover, there is evidence that bacteriophages can modulate cancer cells, which opens up potential therapeutic avenues in oncology [23].

**Is phage present in food products? What type of food or commodity group could be a source of hazardous phage?**

Phages are ubiquitous in natural environments and may be found along the entire food production chain, from farm to fork (Table 1). Their density in water can reach up to 10^8^ PFU/mL, while in soil, it is approximately 1.5 × 10^8^ PFU/g [24]. These high densities indicate that phages significantly maintain bacterial community homeostasis across various ecosystems, including the human body, soil, and marine environments [25].

Red meat, poultry, and vegetables are perishable refrigerated foods with complex microbial ecosystems and significant bacterial populations [26]. Any food category that promotes bacterial development has the potential to harbour phages. However, the prevalence of phages in food is poorly investigated. The source of the food, the techniques used to process it, and the storage conditions can all substantially impact the presence and quantity of phage in the final food product.

Phage contamination has been discussed as a serious issue in the dairy industry since many fermentation processes depend on the ability of bacterial cells to change substrates. However, Mancini et al. [27] examined natural whey starter (NWS) cultures used in Trentingrana cheese production. Six dairy factories were analysed over a year, identifying 120 distinct lytic phages, primarily infecting *Lactobacillus helveticus*. Phage titres ranged from 2 × 10^6^ to 9 × 10^8^ PFU/mL. Despite the high phage presence, the NWS cultures maintained effective acidification, suggesting a balance between phages and bacterial hosts.

Several studies have directly measured phage prevalence in foods through isolation and enumeration. In the research by Thung et al. [28], 29 bacteriophage isolates were obtained and further examined for titre via agar overlay assay, which were determined within the range of 10^8^ to 10^11^ PFU/mL. Premarathne et al. [29] determined bacteriophages within the range of 10^2^ to 10^10^ PFU/mL, and they were most frequently isolated from chicken (60%) samples.

While earlier studies have primarily focused on isolating and quantifying individual phages from foods, more recent approaches have broadened the scope by examining the entire virome (the collection of viral genetic material present in food matrices) to better understand the overall prevalence and diversity of phages and other viruses. Blanco-Picazo et al. [30] studied the viromes of food products. According to VirSorter, the most significant number of viral contigs were derived from viromes of shellfish, followed by spinach. Spinach viromes also included a significant number of phage sequences identified by PHASTER. In another study, they found that phage particles predominated in the viromes of retail food sources, with protein content from phages accounting for 71.8% in fish, 52.9% in mussels, and 78.7% in chicken samples [31].

**Table 1:** Phage quantity in selected environmental and food samples.

Source		Phage Quantity [PFU ^1^/mL or g]	Reference
	Soil	1.50 × 10^7^–1.5 × 10^8^	[24]
	Water	1 × 10^8^	[24]
	Sewage	1.04 × 10^11^–1.14 × 10^11^	[28]
Food	Beef	7.40 × 10^9^–8.10 × 10^10^	[28]
		3.00 × 10^3^–7.00 × 10^8^	[29]
	Chicken	7.27 × 10^10^–1.37 × 10^11^	[28]
		5.3 × 10^3^–9.72 × 10^10^	[29]
	Clam	8.93 × 10^10^	[28]
	Cockles	6.87 × 10^10^–8.33 × 10^10^	[28]
	Shrimp	7.77 × 10^10^–8.90 × 10^10^	[28]
	Whey	2 × 10^6^–9 × 10^8^	[27]
	Cucumber	9.10 × 10^8^–9.90 × 10^8^	[28]
	Lettuce	1.08 × 10^9^	[28]
	Vegetables	4.70 × 10^2^–3.87 × 10^6^	[29]

^1^ PFU—plaque-forming unit.

### 3.2. Hazard and Food—Exposure Assessment

**What are the possible routes of phage exposure (risk pathway)?**

The possible routes of phage exposure concerning the food chain as as follows:

- Consumption of food products may result in exposure if these foods contain phages [31].
- Handling and processing: Cross-contamination can happen while handling and preparing food, possibly transmitting phages into food items. Phage contamination may result from poor sanitation standards or poor hygiene procedures in factories that process foods [32].
- Water contamination: Water used in the preparation and processing of food may include phages. Using the water for cleaning, irrigation, or food preparation might expose people to the phages present in it [33,34].
- Animal contact: Direct and indirect interaction with animals in the food chain, such as cattle and poultry, can spread phages. These animals may serve as phage reservoirs, and exposure can happen if one touches their faeces or body fluids. It is important to remember that the existence of antibiotic-resistant bacteria might result in serious health hazards since they can either cause sickness directly or cause pathogens to acquire antibiotic-resistant features [35].

Other implications for the risk pathway are [36]:

- Biofilm formation: Temperate phages can promote biofilm development by facilitating the aggregation and persistence of bacterial communities. Biofilms, structured consortia of bacteria encased within an extracellular matrix, enhance bacterial survival and resilience in various environments, including food production systems. Temperate phages may strengthen biofilm stability, contributing to bacterial persistence under adverse conditions.
- Mutually beneficial relationships: Temperate phages can establish symbiotic relationships with bacterial hosts, providing adaptive advantages. While phage-mediated gene transfer may enhance bacterial pathogenicity by introducing virulence factors, it can also confer benefits such as improved survival, stress tolerance, and adaptability to fluctuating environmental conditions.

The risk pathway, which means the steps necessary to move from phage infection to disease, can be as follows:

1. Phage introduction: With their source being water, food, or direct contact with infected people or objects [30], phages can enter the body in several ways, e.g., topical, intravenous, transnasal, transrectal, transurinary, or oral routes. However, recent studies and in vitro experiments have shown that phages can translocate from the gut surface to the blood [37].
2. Phage attachment and infection: Phages insert their genetic material into the bacterial cells by adhering to particular receptors on the surface of susceptible bacteria. This starts the process of bacteriophage infection [38].
3. Bacterial replication and lysis: Phages replicate their genetic material and create many phage particles within bacterial cells using the bacterial machinery. Infected bacteria eventually experience lysis (cell break), releasing freshly produced phage particles into the environment [39].

To effectively delineate a risk pathway, it is essential to consider the primary mechanisms implicated in phage-induced lysis. For example, holin proteins are crucial for bacterial cell lysis, particularly in the context of λ bacteriophages. They aggregate in cellular membranes and are responsible for breaking them apart, leading to cell destruction. A notable finding is the strong inverse correlation between the local hydrophobicity of holin proteins and the time it takes for cell lysis to occur. This indicates that higher hydrophobicity is associated with shorter lysis times, highlighting the importance of hydrophobic interactions in disrupting bacterial membranes [40]. Another example is the lysis mechanism of *Shewanella oneidensis* by phage LambdaSo, which involves a combination of four protein factors: a pinholin, a signal-anchor-release (SAR) endolysin, a spanin complex (Rz/Rz1), and a previously uncharacterised protein named Lcc6. This discovery broadens our understanding of the essential components required for phage-induced lysis [41]. Gene transfer and virulence may play a crucial role in phage exposure. The study of Gabashviliet al. [14] covers strong statistical evidence for bacteriophage-mediated intra-species recombination of antibiotic resistance genes (ARGs). This recombination primarily involves genes encoding antibiotic efflux pumps from the major facilitator (MF) superfamily, as well as from the ATP-binding cassette (ABC) and resistance-nodulation-division (RND) families. This was observed in various human pathogenic bacteria, including *Salmonella enterica*, *Staphylococcus aureus*, *Staphylococcus suis*, *Pseudomonas aeruginosa*, and *Burkholderia pseudomallei*. This finding indicates that bacteriophages can promote genetic diversity and spread resistance traits across different genera.

**Is it possible that phages enter the food chain because of accidental release, and if so, how much does this happen?**

Insights into phage genetics have enabled the creation of fundamental tools that remain essential in recombinant DNA technology. Moreover, phage-based methods such as display and typing have become standard practices in molecular biology laboratories and have been adapted for various additional applications. For instance, bacteriophages are now employed as delivery systems for protein and DNA vaccines [42].

Although most bacteriophages are classified as risk group 1, posing no threat to human health, control measures should still be implemented to minimise their spread and protect the environment. Bacteriophages serve as significant reservoirs of genes that can be transferred between bacteria, and the release of recombinant phages into the environment could substantially affect nearby susceptible bacterial populations [42].

Moreover, unintended exposure can occur during the evaluation of phage biocontrol effectiveness, due to leftover phages that might affect the recovery and counting of surviving bacteria on the food surface or food contact surfaces after treatment. Typically, the efficacy of a phage formulation is tested by applying it to surfaces that have been pre-inoculated with the target pathogen and incubating the treatment therapy for a set period. The food sample is transferred into a liquid buffer and stomached to release the surviving bacteria for their enumeration. During these final steps, there is a potential for residual phage to interact with bacterial survivors, which could affect the calculated efficacy of the phage. Limited studies demonstrated that bacterial reductions in these experiments occur specifically during treatment and not during sample recovery [43].

**Is the phage sensitive/resistant to food processing techniques? And how do food processing techniques influence phage?**

Phages can react differently to food processing methods. The particular phage strain, processing conditions (temperature, pH, duration), and treatment used all impact how food processing affects phages [44]. Many food processing techniques may detrimentally affect phages by reducing their quantity or making them inactive [32]. Heat treatments, such as pasteurisation, can inactivate some phages. Lactic acid bacteria (LAB) phages have demonstrated minimal resistance to both low-temperature, long-duration and high-temperature, short-time pasteurisation procedures, which are thermal treatments used to eliminate harmful bacteria or extend the shelf life of dairy products. The International Dairy Federation recommends 90 °C exposure for over 15 min to eradicate phages, even though most phages cannot survive 90 °C for longer than 2 min. Some phages may, however, possess defences or modification mechanisms that make them more resistant to particular processing methods [45]. Several different chemical and physical sanitisation techniques are used in the food industry, and the efficiency with which they inactivate phages varies. For instance, by causing denaturation of their proteins and genetic material, heat treatments given to food products can effectively destroy or reduce phage populations. In addition, high-pressure processing, UV light treatment, and specific chemical treatments can affect the survivability of phages in conditions associated with food [46]. Given phages’ inherent diversity and unpredictability, food producers must consider several aspects to control them efficiently. These include phage concentration, certain phage traits, and the effectiveness of processing methods in phage control. Regular monitoring and validation of processing techniques are essential to guarantee that phage populations are adequately controlled and the necessary food safety and quality criteria are maintained. Food producers can reduce the risks connected with phage infection and ensure the creation of safe and efficient food products by considering these variables [47].

In the work of Tomat et al. [48], phage viability was tested under refrigeration, high temperatures, various salt concentrations, and pH levels typical of meat and dairy products. Phages were fully inactivated at 90 °C, though DT2 and DT6 showed some resistance, surviving 2 min at this temperature. It appeared that tris-magnesium gelatin buffer best protected phages against heat. Viability was slightly or moderately reduced at 63 °C and 72 °C. Cations and low water activity (0.90 and 0.95) did not affect viability. All six phages tolerated pH treatments, showing greater resistance to alkaline conditions up to pH 11. Overall, phage activity was only partially affected by low temperature, high Na^+^ concentration, and low pH, remaining viable and infectious against three pathogenic *E. coli* strains in most tested conditions.

### 3.3. Hazard Characterisation

**Do phages cause illness in humans?**

The widespread and regular presence of phages provides validity to the idea that they are harmless to people and the environment and may be effectively used in agriculture [49].

However, studies have already indicated a possible association between bacteriophages and Parkinson’s disease (PD) [50]. Recent research by Zhao et al. [48] has shown that changes in gut phage populations have been observed in PD patients, affecting dopamine-producing bacteria and gut permeability. Studies also found an imbalance of viruses in the substantia nigra (SN) of PD patients, with specific phages potentially disrupting genes involved in dopamine production, which may contribute to PD development. Notably, three specific phages—*Proteus* phage VB-PmiS-Isfahan, *Escherichia* phage phiX174, and *Lactobacillus* phage Sha1—were identified in the SN and are thought to impair neural gene expression and weaken immune responses in the brain. This groundbreaking research highlights that phages might influence PD pathogenesis, offering a new perspective on the role of brain microbiota in the disease [51].

**What are the potentially harmful properties of the phages? Do phages have virulence genes?**

Phage particles usually have a protein covering that might contain lipids, DNA, or RNA, which can have a length of a few thousand to hundreds of thousands of base pairs and make up the genetic material inside this coat. Additionally, phages include genes crucial to their replication and capacity to infect host cells. These genes often have a role in the phage’s structural protein production, facilitating the identification of host cells and promoting DNA replication during infection [52].

Although phages may carry virulence-associated genes, their presence does not inherently pose a direct risk to human health. Phages can mediate a process known as phage conversion, whereby they transfer genetic material, including virulence factors, to their bacterial hosts. This genetic exchange can enhance the pathogenic potential of previously non-pathogenic strains, potentially transforming them into virulent organisms if the transferred genes encode toxins, adhesion factors, or other pathogenic traits. Converting phages, primarily temperate rather than strictly lytic, have been implicated in harbouring a range of virulence determinants found in bacterial pathogens affecting plants, animals, and humans [53].

Within the food chain, the presence of bacteriophages carrying virulence genes raises concerns about the possible transfer of these genes to bacterial pathogens, thereby enhancing their virulence or pathogenicity. For example, *Bacillus cereus* bacteriophages may increase in fermented foods where *B. cereus* is naturally present [54].

The impact of phage-borne virulence genes ultimately depends on multiple factors, including the host bacterium’s susceptibility to phage infection and the specific genetic content of the phage. Importantly, not all bacteriophages carry virulence factors; their presence is highly strain-dependent.

**What are the modes of pathogenicity associated with phages?**

Bacteriophages can contribute to bacterial pathogenicity through the following mechanisms [55]:

1. Lysogenic conversion: Occurs when a phage integrates its genetic material into the bacterial genome, establishing a lysogenic relationship with the host. The expression of phage-derived genes can alter bacterial phenotypes, including the synthesis of virulence factors or toxins, thereby enhancing pathogenic potential. Beyond pathogenicity, lysogenic phages also have potential applications in biotechnology, such as improving fermentation processes in food production and agriculture [56].
2. Transduction: Allows phages to mediate horizontal gene transfer (HGT) between bacterial cells, facilitating the movement of genetic material, including virulence genes, across populations. This transfer can increase the virulence of recipient strains or contribute to the emergence of novel pathogens [57].
3. Bacterial cell lysis: This leads to the release of endotoxins and pro-inflammatory molecules. These components may exacerbate immune responses in the host, potentially worsening existing infections or intensifying disease symptoms [58].
4. Presence of virulence factors (VFs): This has been demonstrated in specific bacterial populations. In *Streptococcus pyogenes*, for example, strains exhibiting evidence of HGT were found to carry specific phage-derived virulence determinants, which were absent in non-HGT strains. This finding underscores the role of phage-mediated gene transfer in transmitting pathogenic traits.
5. Phylogenetic analysis: This revealed that virulence factors through HGT occurred independently across diverse *S. pyogenes* strains, suggesting that phage activity has contributed to multiple, distinct evolutionary pathways.
6. Synteny analysis demonstrated a random organisation of genetic elements within HGT-positive strains, likely reflecting phage-associated genes’ stochastic integration and retention. Such genomic rearrangements may further influence bacterial fitness and virulence.

Collectively, these findings highlight the critical role of phage-mediated HGT in enhancing bacterial pathogenicity and underscore the importance of understanding phage–host interactions in the context of bacterial evolution and disease dynamics [55].

**Can any modification/manipulation of phages be harmful to humans?**

Safety is paramount when modifying or engineering bacteriophages for biotechnological or therapeutic applications. While lytic proteins encoded by phages have demonstrated efficacy against multidrug-resistant Gram-positive infections and are generally considered safe for therapeutic use, genetic manipulation introduces potential risks that must be carefully evaluated [59].

Genetic modification of phages, particularly efforts to expand host range or enhance bactericidal activity, may result in unintended consequences, including increased virulence, altered interactions with human cells, or unforeseen effects on the immune system. Such outcomes could compromise human health if not correctly anticipated and mitigated [60].

Therefore, any genetic engineering of phages intended for medical or industrial deployment must undergo comprehensive safety evaluations. These assessments should rigorously identify and characterise potential hazards associated with the modified phages, ensuring that genetic alterations do not introduce unacceptable risks. Implementing stringent regulatory oversight, meticulous testing protocols, and thorough risk assessments is essential to safeguard the development and application of engineered phages for therapeutic and biotechnological purposes [61].

**Do interactions between phages and infected pathogens pose any risk to humans?**

The interactions between bacteriophages and pathogenic bacteria can have profound implications for human health. Several mechanisms can contribute to this impact, as follows:

- Toxin production: Certain phages carry genes encoding virulence factors, including toxins, which can be expressed following infection of bacterial hosts. These phage-encoded toxins can enhance bacterial pathogenicity and contribute to human disease severity [62]. Additionally, phage-encoded loci, specific genetic elements introduced during infection, can modulate bacterial antigenicity, toxicity, and metabolic capabilities, thereby altering bacterial traits and behaviour [63].
- By-products and immune response: Replicating phages within bacterial hosts and subsequent lysis release bacterial components such as endotoxins and other pro-inflammatory molecules. These by-products can provoke immune responses, potentially exacerbating pre-existing infections and intensifying disease symptoms. Furthermore, suppressing pro-inflammatory mediators during phage–bacteria interactions may impair adequate bacterial clearance by phagocytes. Extensive evidence indicates that lytic bacteriophages significantly influence mammalian immune systems, a phenomenon particularly relevant in phage therapy [64].
- Genetic exchange and antibiotic resistance: Phages facilitate horizontal gene transfer among bacterial populations, including antibiotic resistance genes. This genetic exchange poses a substantial public health concern, contributing to the emergence and spread of antibiotic-resistant infections [65].

**What is the impact of phages on the genomic structure of the gut microbiome?**

A varied population of microorganisms, like bacteria, viruses, and phages, shapes the gut microbiome. The phages can selectively target and infect microorganisms in the stomach. This selective target from phages can modify the genomic makeup of bacterial populations in the gut microbiome and cause genetic changes through their interactions with bacteria [66].

Beyond direct bacterial lysis, phage activity within the gastrointestinal tract can have broader implications by altering the composition and stability of the gut microbiota, which in turn may affect host health. Changes in the gut microbiota may reveal an impact on host health that can result from phages infecting and lysing bacteria. This may involve changes to the number, variety, and composition of bacterial species in the gut [12]. Moreover, the host’s health may be impacted by changes in the gut microbiota brought on by phage infections. Digestion, food absorption, immune system function, and disease susceptibility are just a few of the elements of human health that can be impacted by changes in the gut microbiome, microbial balance, and genetic composition [67].

Since many tail-bearing bacteriophages exist in the human gut, transduction may play a substantial role in HGT in the gut microbiota. This indicates that phages can transmit genetic material across bacteria, including genes linked to virulence or antibiotic resistance. Such gene transfers may significantly alter bacterial populations’ genomic makeup and characteristics, perhaps affecting their antibiotic resistance and pathogenicity profiles [68].

### 3.4. Summary

Table 2 summarises questions and answers to develop a risk profile of bacteriophages in the food chain as an emerging hazard.

**Figure 1:**
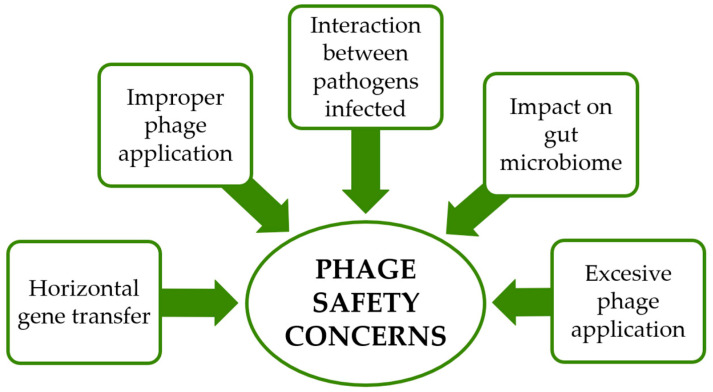
The primary safety concerns associated with phages in the food industry.

Meanwhile, Figure 1 presents the primary safety concerns associated with using phages in the food industry.

The expected survival, multiplication, and dissemination of phages in the food chain raise important considerations regarding their interactions with organisms and potential harm to humans. While phages are primarily known for targeting bacterial pathogens, their complex interactions with eukaryotic cells and the food environment necessitate thoroughly examining their safety and efficacy.

The primary safety concerns associated with using phages in the food industry involve the potential for horizontal gene transfer, particularly the dissemination of antibiotic resistance genes between bacterial populations. Although such events are context-dependent and relatively infrequent, they remain a significant area requiring further investigation. Additionally, improper or excessive phage application, such as use at suboptimal stages of food processing, may promote the emergence of phage-resistant bacterial strains, thereby reducing the long-term effectiveness of phage-based interventions.

#### 3.4.1. Area of Hazard and Food in the Risk Profile

Although phages are frequently ingested through food, the potential adverse effects of direct phage consumption on human health remain unclear. Nevertheless, the presence of phages in the food chain does not always directly endanger human health. The quantity of phages consumed can vary considerably depending on factors such as the type of food product, the level of phage contamination, and individual dietary habits.

The properties of the phages and bacteria, the host’s immunological reaction, and the host’s general health can all impact the multidimensional pathway from phage infection to illness. It is essential to consider these variables as they play a significant role in determining the potential risks associated with phage infection leading to disease. The risk pathway from phage infection to disease involves steps that facilitate the transition from bacteriophage activity to the emergence of pathogenic traits in bacterial hosts. This pathway encompasses the mechanisms of phage lysis, gene transfer, and the subsequent development of virulence in bacteria.

Phage-mediated recombination plays a significant role in the emergence and transmission of multidrug resistance across a broad spectrum of bacterial species and genera, including human pathogens. This highlights the importance of understanding bacteriophage interactions in the context of public health and antimicrobial resistance.

While bacteriophages are naturally ubiquitous in the environment and commonly ingested through food without adverse effects, the unintended release of concentrated or genetically modified phages requires special attention. Although phages are widely recognised for their antimicrobial properties and safety in food applications, accidental environmental releases, particularly of engineered or high-titre preparations, could potentially disrupt microbial ecosystems or facilitate horizontal gene transfer events. These ecological impacts, while theoretical, underscore the need for strict biosafety measures during phage production, handling, and application.

#### 3.4.2. Area of Evaluation of Adverse Health Effects in the Risk Profile

Although bacteriophages are generally considered harmless to humans and have been explored as potential alternatives to antibiotics, there is no clear consensus regarding the specific diseases, if any, that phages may cause in humans. Theoretically, particular phages could disrupt the human microbiota by infecting beneficial bacterial populations. While the extent and frequency of such disturbances remain poorly characterised, potential shifts in microbiota composition could have implications for human health [69].

Phages can influence the abundance and diversity of specific bacterial species within the gut microbiome. Phages can selectively target certain bacterial strains through their infection and replication cycles while sparing others. This selective pressure can lead to shifts in the relative abundance of bacterial species, altering the overall genomic structure of the gut microbiome.

The impact of phages on the genomic structure of the gut microbiome is profound, influencing both the diversity and functionality of microbial communities. Phages, as key components of the gut virome, engage in genetic exchanges with bacterial hosts, leading to significant structural variations in the gut microbiome. This interplay is crucial for understanding microbial dynamics and health outcomes.

Up to now, the main risk question can be stated as follows: Do phages in the food chain pose a risk to human health? Current evidence suggests that any health risks associated with phage consumption or their disease-causing behaviour are limited. However, a significant knowledge gap remains due to the lack of prevalence data on phages and exposure assessment.

## 4. Conclusions

Although numerous studies have demonstrated the safety and efficacy of phage application within the food chain, ongoing monitoring remains essential. It is important to recognise that zero risk does not exist; as biological agents, phages must be continuously evaluated for potential adverse effects on human health in both food and clinical contexts. In particular, the responses of susceptible populations to phage exposure should be carefully examined.

To our knowledge, this is the first bacteriophage risk profile performed according to the methodology recommended by FAO/WHO and EFSA. Therefore, it can support risk managers in the next step during the risk management process in the food chain.

## Figures and Tables

**Table 2:** Questions to be considered in the risk profile of bacteriophages in the food chain, along with possible answers.

Stage of Risk Assessment	Question	Answer/Risk Profile
Hazard and Food		
Hazard identification	What is the biology of bacteriophages?	Viruses have the unique capacity to infect and spread throughout bacteria.
	Are phages present in food products?	Yes, within the range of 10^2^ to 10^11^ PFU ^1^/mL.
	What type of food or commodity group could be a source of hazardous phages?	Any food category that promotes bacterial development has the potential to harbour phage. However, the prevalence of hazardous phages in food is scarcely researched.
Exposure assessment	What are the possible routes of phage exposure (risk pathway)?	Consumption of contaminated food Handling and processing Water contamination Direct and indirect interaction with animals
	Is it possible that phages enter the food chain because of accidental release, and if so, how much does this happen?	Unintended exposure can occur during any manipulation in the laboratory and/or when phage biocontrol efficacy testing due to residual phages affecting the recovery and counting of surviving bacteria on food or contact surfaces.
	Are phages sensitive/resistant to food processing techniques? And how do food processing techniques influence phages?	Phages can react differently to food processing methods. The particular phage strain, processing conditions (temperature, pH, duration), and treatment used all impact how food processing affects phages.
Evaluation of Adverse Health Effects		
Hazard characterisation	Do phages cause illness in humans?	The first research has indicated a possible association between bacteriophages and Parkinson’s disease.
	What are the potentially harmful properties of phages? Do phages have virulence genes?	Phages could have virulence genes, but it does not always mean they are dangerous to people.
	What are the modes of pathogenicity associated with phages?	Lysogenic conversion Transduction Bacterial cell lysis Presence of virulence factors
	Can any modification/manipulation of phages be harmful to humans?	It is critical to consider the safety consequences before genetically modifying phages for medicinal reasons or increasing their capacity to infect various bacteria.
	Do interactions between phages and infected pathogens pose any risk to humans?	Toxin production By-products and immune response Genetic exchange and antibiotic resistance
	What is the impact of phages on the genomic structure of the gut microbiome?	Modification of the genomic makeup of bacterial populations in the gut microbiome

^1^ PFU—plaque-forming unit.

## Data Availability

No new data were created or analyzed in this study. Data sharing is not applicable to this article.

## Abbreviations

The following abbreviations are used in this manuscript:

IBD	Inflammatory bowel disease
PD	Parkinson’s disease
SN	Substantia nigra
VRFC	Viral RNA fragment counts
VPGs	Virus-correlated PD-related genes
VFs	Virulence factors
HGT	Horizontal gene transfer
SAR	Signal-anchor-release
ARGs	Antibiotic resistance genes
MF	Major facilitator
ABC	ATP-binding cassette
RND	Resistance-nodulation-division
STEC	Shiga toxigenic *E. coli*
GRAS	Generally Recognised As Safe
FDA	Food and Drug Administration
PFU	Phage-forming units
EPEC	Enteropathogenic *E. coli*
a_w_	Water activity
SVs	Structural variations
